# Temporal trends in artemisinin partial resistance and other antimalarial drug mutations in *Plasmodium falciparum* from Kagera region, Northwestern Tanzania, 2021–2023

**DOI:** 10.3389/fgene.2026.1776108

**Published:** 2026-06-03

**Authors:** Alfred Simkin, Salehe S. Mandai, Abebe A. Fola, Neeva Wernsman Young, Jacob Marglous, Dativa Pereus, Catherine Bakari, Rashid A. Madebe, Misago D. Seth, Rule B. Mrengela, Angelina J. Kisambale, Gervas A. Chacha, Celine I. Mandara, Filbert Francis, Daniel Mbwambo, Issa Garimo, Frank Chacky, Sijenunu Aaron, Abdallah Lusasi, Fabrizio Molteni, Ritha J. A. Njau, Stella Kajange, Samwel L. Nhiga, Ally Mohamed, Jonathan J. Juliano, Deus S. Ishengoma, Jeffrey A. Bailey

**Affiliations:** 1 Department of Pathology and Laboratory Medicine, Brown University, Providence, RI, United States; 2 Ifakara Health Institute, Dar es Salaam, Tanzania; 3 Center for Computational Molecular Biology, Brown University, Providence, RI, United States; 4 National Institute for Medical Research, Dar es Salaam, Tanzania; 5 National Malaria Control Programme, Dodoma, Tanzania; 6 Swiss Tropical Public Health Institute, Dar es Salaam, Tanzania; 7 Department of Parasitology and Medical Entomology, Muhimbili University of Health and Allied Sciences, Dar es Salaam, Tanzania; 8 President’s Office, Regional Administration and Local Government, Dodoma, Tanzania; 9 Division of Infectious Diseases, University of North Carolina School of Medicine, University of North Carolina at Chapel Hill, Chapel Hill, NC, United States; 10 Curriculum of Genetics and Molecular Biology, University of North Carolina School of Medicine, University of North Carolina at Chapel Hill, Chapel Hill, NC, United States; 11 Institute for Global Health and Infectious Diseases, University of North Carolina School of Medicine, University of North Carolina at Chapel Hill, Chapel Hill, NC, United States; 12 Department of Epidemiology, Gillings School of Global Public Health, University of North Carolina at Chapel Hill, Chapel Hill, NC, United States

**Keywords:** artemisinin, genomics, kelch, malaria, Plasmodium falciparum, resistance, surveillance, Tanzania

## Abstract

Artemisinin-based combination therapies (ACTs) remain the cornerstone of malaria treatment, yet the emergence of artemisinin partial resistance (ART-R) in Africa threatens their efficacy. ART-R is primarily associated with mutations in *Plasmodium falciparum* kelch13 (K13), notably R561H, which has been linked to delayed parasite clearance in East Africa. We genotyped 2,866 *P. falciparum* isolates from seven districts in Tanzania’s Kagera region (2021–2023) using 121 molecular inversion probes (MIP) targeting key resistance loci to characterize trends in ART-R and other resistance markers. The WHO-validated K13 mutation R561H persisted in border districts of Karagwe and Kyerwa, with prevalence ranging from 14% to 26%, and appeared for the first time in Muleba in 2022 (10.0%) and Bukoba rural district (0.7%) in 2023, indicating eastward spread toward Lake Victoria. Regional prevalence of R561H rose from 5.5% in 2021 to 6.9% in 2023. Additional validated (A675V) and candidate (V568G, P441L) mutations were detected at low frequencies. Markers associated with reduced sensitivity to partner drugs showed minimal change. Early DHFR and DHPS mutations were near fixation and high-level resistance markers (DHFR I164L and DHPS A581G) exhibited marked gradients. These latest mutations are significantly spatially colocalized (weighted spearman R^2^ = 0.58, P = 0.045) and co-occur within a number of individual genomes. These results highlight notable variation in mutation prevalence and underscore the importance of high-resolution surveillance to identify emerging hotspots and guide targeted interventions. Sustained molecular monitoring is critical to inform treatment policy, preserve ACT efficacy, and mitigate the risk of widespread resistance across East Africa.

## Introduction

Africa is facing a looming public health crisis with the emergence of artemisinin partial resistance (ART-R) (Rosenthal et al., 2024). ART-R is associated with mutations in *Plasmodium falciparum* kelch13 (K13), which have been linked to prolonged parasite clearance and longer clearance half-lives. Multiple World Health Organization (WHO) validated and candidate K13 polymorphisms, including P441L, C469Y/F, R561H, R622I, and A675V, are now detected widely in most countries along the Great Rift Valley (Rosenthal et al., 2024). Another focus of P441L has also emerged in Southern Africa around the Namibia-Zambia border (Eloff et al., 2025; Martin et al., 2025). Mutations in partner drug resistance will be critical in clinical failure of artemisinin combination therapies (ACT), as was seen in Southeast Asia (Björkman et al., 2024). As ACTs rely on artemisinin to reduce parasite burden, resistant parasites are increasingly exposed to partner drugs as monotherapy, heightening the risk of resistance development (Rosenthal et al., 2024). Additionally, existing partner drug resistance mutations in Africa could further accelerate the spread of ART-R, mirroring the pattern observed with mefloquine resistance in Asia (Björkman et al., 2024). Understanding the spread of ART-R and partner drug mutations requires samples collected across multiple years in affected areas. This information is essential for malaria control planning in order to implement potential interventions that may reduce resistance, such as multiple first-line therapy, triple or sequential ACT, non-ACT antimalarial drugs, or potentially malaria vaccination.

The Kagera Region in Northwest Tanzania appears to be part of the outward spread of the K13 R561H mutation from a likely origin in Rwanda. After initial detection of the polymorphism in Rwanda in samples collected in 2014, a therapeutic efficacy study (TES) in 2018 confirmed clinical ART-R (Uwimana et al., 2020; Uwimana et al., 2021). Subsequent analysis of samples collected from asymptomatic individuals in the 2014 Rwanda Demographic Health Survey showed the mutation occurring along the border with Tanzania (Kirby et al., 2023). In 2021, the R561H mutation was found in multiple clinics in Kagera along the Rwanda border, with a prevalence of 22.8% [31 of 136] in Karagwe, 14.4% [17 of 118]) in Kyerwa, and 1.4% [two of 144] in Ngara (Juliano et al., 2024). A TES in the same region in 2022 documented clinical ART-R, based on delayed clearance with the median parasite clearance half-life in patients harbouring parasites with R561H mutation reaching over 6 h in all treatment groups, further confirming its clinical impact (Ishengoma et al., 2024).

Beyond ART-R, the efficacy of other antimalarials is at risk. Non-synonymous polymorphisms in *P. falciparum* multidrug resistance (MDR1) protein and *P. falciparum* chloroquine resistance transporter (CRT) have been linked to reduced susceptibility to lumefantrine (Venkatesan et al., 2014) and amodiaquine (Humphreys et al., 2007). Markers for lumefantrine resistance remain unclear (Rosado et al., 2024), but the wild type MDR1 N86Y allele has been associated with re-infection following artemether-lumefantrine (AL) treatment (Sisowath et al., 2005). As lumefantrine and amodiaquine are the most widely used ACT partner drugs across Africa, monitoring genetic mutations linked to reduced susceptibility to the partner drug is crucial. Mutations in *P. falciparum* dihydrofolate reductase (DHFR) and *P. falciparum* dihydropteroate synthase (DHPS) impact susceptibility to pyrimethamine (P) and sulfadoxine (S), respectively. Mutations conferring high-level resistance to SP, a combination used frequently in chemoprevention, are also important to monitor, in particular DHFR N51I, C59R, S108** N/T**, and I164L, which together form the DHFR **IRNL** resistance haplotype, and DHPS A437G, DHPS K540**E**, and DHPS A581G, which together form the DHPS **GEG** resistance haplotype. In each of these genes, these haplotypes have formed through sequential mutations, with the latest mutations of DHFR I164L and DHPS A581G demonstrating the greatest pyrimethamine and sulfadoxine resistance, respectively (Hastings et al., 2002; Mousa et al., 2025).

To understand the dynamics of ART-R and associated partner drug mutations in this emerging hotspot, we sequence samples from cross-sectional surveys within the ongoing malaria molecular surveillance (MMS) in Tanzania’s Kagera region from 2021 to 2023. By analyzing temporal and spatial trends of validated and candidate K13 mutations alongside markers for partner drug tolerance (CRT K76T, and MDR1 N86Y) and antifolate resistance (DHFR I164L, DHPS A581G), this study provides critical insights into the evolution and spread of antimalarial drug resistance in the region. These findings are essential for informing malaria control strategies, guiding drug policy decisions, and supporting interventions—such as multiple first-line therapies, triple ACT regimens, future deployment of non-ACT antimalarial drugs, or integration with malaria vaccination—to preserve the efficacy of current treatments and mitigate the threat of widespread resistance.

## Methods

### Ethics statement

This study was conducted as part of the Molecular Surveillance of Malaria in Tanzania (MSMT) project, whose protocol was submitted to, reviewed, and approved by the Medical Research Coordinating Committee (MRCC) of the National Institute for Medical Research (NIMR) in Tanzania (NIMR/HQ/R.8a/Vol.IX/3,579). All research participants provided individual consent (or assent for children aged 7–17 years) for both participation in the survey and biobanking for future research. For participants under the legal age of adulthood in Tanzania (<18 years), consent was obtained from a parent or guardian. The informed consent form was developed in English, translated into Kiswahili, and used to obtain consent verbally and in writing. Participants either signed the consent or assent form or, if illiterate, provided a thumbprint accompanied by the signature of an independent witness.

### Patient samples

The MSMT project collected capillary dried blood spot (DBS) samples from patients aged 6 months and older who tested positive for malaria via rapid diagnostic tests at participating clinics. The 2021 and 2022 surveys covered 100 health facilities across ten regions: Dar es Salaam, Dodoma, Kagera, Kilimanjaro, Manyara, Mara, Mtwara, Njombe, Songwe, and Tabora while the 2023 survey covered all 26 regions of Mainland Tanzania; here, we focus only on clinics in the Kagera region. The sites in Kagera were selected to be able to evaluate the border region with Rwanda, where K13 polymorphisms were previously described, as well as to assess if spread was occurring to the East toward Lake Victoria and South toward Lake Tanganyika. Samples were collected between 01 February and 26 July, 01 February and 03 August, and 23 January and 28 August in 2021, 2022, and 2023, respectively. In 2021, one site in each district was sampled in Biharamulo, Karagwe, and Misenyi districts, while two sites were covered in Bukoba, Kyerwa, Muleba, and Ngara. In 2022, one site in each of 3 districts was sampled (Karagwe, Muleba, and Ngara). In 2023, one site in each district was sampled in Biharamulo, Bukoba, Karagwe, and Misenyi districts, while two sites were covered in Kyerwa, Muleba, and Ngara. Written informed consent was obtained as approved by the Tanzanian Medical Research Coordinating Committee of NIMR. Deidentified dried blood spot (DBS) samples from 2021 were processed at Brown University, and 2022 and 2023 were processed at the National Institute for Medical Research (NIMR) in Dar es Salaam, Tanzania.

## DNA extraction and sequencing

DNA was extracted using the Chelex-Tween protocol, followed by molecular inversion probe (MIP) capture. The resulting library was purified using the New England Biolabs (NEB) Gel extraction kit (NEB Inc., Ipswich, MA, United States). The NEBNext® Library Quant Kit for Illumina® (NEB Inc., Ipswich, MA, United States) was used for qPCR-based library quantitation and sequenced using Illumina platforms, MiSeq and NextSeq (Illumina, Inc., San Diego, CA, United States), using 3D7 and 7G8 as controls (MRA-102G and 152G, BEI Resources, Manassas, VA). The sequencing reads were then transferred to the main server for downstream bioinformatics analysis. Libraries lacking sufficient read depth were rebalanced and resequenced.

### Parasite genotyping

We attempted sequencing of 572 *Plasmodium falciparum* isolates from 3 districts in 2022 and 1,414 isolates from 7 districts in 2023. Samples collected in 2021 were previously reported (Juliano et al., 2024) and were genotyped using the full drug resistance MIP panel comprising 815 probes. For the 2022 and 2023 collections, parasites were genotyped using smaller targeted subsets of this panel designed to capture key antimalarial resistance loci. The 2022 panel included 121 probes, while the 2023 panel expanded coverage to 184 probes. These probes target five major resistance-associated genes: *pfk13, pfmdr1*, *pfcrt*, *pfdhfr,* and *pfdhps*, as well as *plasmepsin2 and plasmepsin3*. Probes were primarily designed to capture known resistance-associated codons and functional domains within these genes, with a tiled design across the full coding region of *pfk13* to enable detection of both established and emerging variants. To ensure comparability across years, we restricted analyses to genes covered by the smallest shared panel of 121 probes. The details of the genomic regions covered by these 121 probes are included in Supplementary Table S1.

### Bioinformatics analysis

Variant calling was performed using MIPTools to process fastq data, with variants identified through FreeBayes as described previously (Juliano et al., 2024). Downstream filtering followed the same process used for the 2021 dataset, incorporating unique molecular index (UMI) clustering to correct sequencing errors and improve confidence in variant calls and quantification. For known drug resistance genes, we required at least three UMIs covering the genomic region and one UMI containing the mutant allele, while for novel mutations, we required 10 UMIs covering the genomic region and three UMIs containing the mutant allele.

In order to investigate copy number variation (CNV), we used a MIP UMI count normalization method. For each gene, samples were filtered to those with a minimum median UMI count over the probes targeting the gene of 25. Probe UMIs were then normalized to their sample-and-gene median, and these corrected probe UMI values were normalized again to the median corrected value of the probe across samples. As an additional quality filter, samples with a within-sample standard deviation of barcode counts greater than four times the standard deviation of sample-average barcode counts were removed from the final CNV dataset. Per gene, potential copy number-variant samples were identified as having outlier mean corrected-UMI counts, using a normalized fold-change of 1.75.

### Data analysis

Descriptive statistics were used to summarize sample characteristics, including frequencies and proportions of successfully genotyped isolates across years and districts. Prevalence estimates and 95% confidence intervals (CIs) (Clopper-Pearson estimate in proportion_confint method in statsmodels python package (v.0.14.4)) were calculated for key mutations associated with antimalarial drug resistance, stratified by year and district. Mutation prevalences were illustrated with geographical maps. Spatial patterns of resistance mutations were analyzed by the district to detect geographic emergence and expansion. All data visualizations, including maps and mutation trend plots, were generated using the ggplot2 and sf R packages; map shapefiles were obtained from GADM.org. Statistical analyses were conducted using R version 4.2.1 (The R Foundation for Statistical Computing, Vienna, Austria) and Python version 3.14.3. To assess temporal changes across Kagera in key mutation frequencies over the study period, while accounting for differences in sampling across districts, a generalized linear mixed effects model was fit, using a binomial distribution and logit link function, to estimate an overall trend across the region over the study period with sampling year as a fixed effect and district as a random intercept. The model was implemented using the lme4 package in R. To assess district-level trends over the study period, Fisher’s exact tests were performed on mutation frequencies from 2021 to 2023 using the scipy package in Python. For district trends p-values <0.05 following Bonferroni correction for tests over the 7 sampled districts were considered statistically significant.

For analysis, we leveraged previously reported genotyping data from 942 successfully genotyped samples from Kagera from 2021. Prevalences were calculated at the district level. Because genotyping success varied by locus, unweighted prevalence estimates were calculated using locus-specific denominators (*p* = *x*/*n* × 100, where *p* is the prevalence, *x* is the number of samples with mutant alleles and *n* is the number of successfully genotyped samples). Unweighted prevalence was calculated using the miplicorn R package v.0.2.90 (https://github.com/bailey-lab/miplicorn). Maps were generated using the sf package in R 4.2.1.

## Results

### Characteristics of successfully genotyped samples

**FIGURE 1:**
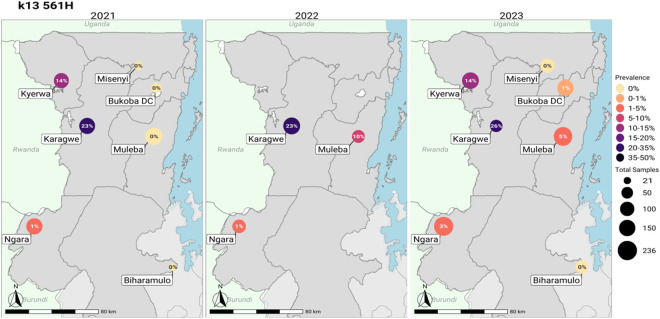
Spatial Distribution and Temporal Changes in K13 R561H. Prevalences are shown as percentages (inside of circles) and as a color scale from light (low prevalence) to dark (high prevalence), while sample counts are represented by point size. Data is aggregated by district.

Overall, 2,979 Kagera samples were collected and sequenced over the 3 years. Of the attempted samples, 2,866 (96.2%) of isolates passed filters providing usable genotyping. Overall, 534 isolates from 2022 to 1,390 isolates from 2023 passed filtering and were compared to 942 previously published isolates from 2021 (Juliano et al., 2024). Sampling in the Kagera region was at 11 sites representing seven districts in 2023, with interim sampling in 2022 of only three sites in three districts (Figure 1). Counts of successfully sequenced samples by district and year are shown in Supplementary Figure S1.

### ART-R K13 polymorphisms

K13 R561H mutations were commonly found from 2021 to 2023, with an average district prevalence across the Kagera region rising over 2 years from 5.5% in 2021 to 6.9% in 2023. Linear modeling of overall R561H frequency across all districts indicated an upward trend that did not quite reach significance (annual odds ratio 1.16; 95% CI [0.94, 1.43])) (Table 1). Newly detected R561H occurred in two additional districts in the East in 2023 compared to 2021, consistent with further detectable spread: The detection in Muleba district was a significant increase, 0% (0/171; 95% CI 0.0%–2.1%) to 5% (11/220; 95% CI 2.5%–8.8%) (Fisher’s exact test, *p = 0.003, Bonferroni p = 0.021*), whereas R561H Bukoba district was an insignificant rise, 0% (0/35, 95% CI 0.0%–10.0%) to 0.7% (1/147, 95% CI 0.0–3.7) (Fisher’s exact test, *p =* 1.0). The only other validated ART-R K13 mutations to be consistently found across all 3 years were mutants A675V and V568G (Table 1). Overall there was no significant increase in prevalences of these mutations across Kagera (A675V year-over-year OR: 3.19, CI [0.90, 11.25]; V568G year-over-year OR: 0.35, CI [0.13, 0.96]). The A675V isolate increased from 0.7% to 4.8% in Karagwe (Fisher’s exact test, *p = 0.09*, Bonferroni *p = 0.63*) and was detected at low prevalence in Muleba and Kyerwa in 2023 (Table 1). The candidate V568G mutation was found in Bukoba in 2021 and 2023, and also in Ngara in 2021 and 2022, and in Kyerwa in 2021 (Table 1). Candidate mutation K13 R622I, known to be at high prevalence in Eritrea and Ethiopia (Fola et al., 2023; Mihreteab et al., 2023), was found once (1/101) in Misenyi in 2023 (Table 1). The candidate mutation K13 P441L, found commonly in Zambia, Namibia, Ethiopia, Rwanda and Uganda (Conrad et al., 2023; Eloff et al., 2025; Martin et al., 2025; Wernsman Young et al., 2025), was found consistently in Kyerwa, and in 2023 in Misenyi (Table 1). In addition, we observed ten other validated or candidate WHO ART-R K13 mutations sporadically at very low prevalences ranging from 0.1% to 0.6% within Kagera (Table 1). Nine other validated and candidate K13 mutations were never observed (Supplementary Table S2). Several non-synonymous K13 mutations of undetermined significance were also identified, with more of these mutations occurring in 2022 and 2023 than in 2021 (Supplementary Table S3 **;** Supplementary Figure S2). These mutations were found within Bukoba, Karagwe, Kyerwa, Misenyi, and Ngara.

**TABLE 1:** District level prevalence of validated and candidate K13 polymorphisms 2021–2023.

Mutation	Year	Biharamulo		Bukoba DC		Karagwe		Kyerwa		Misenyi		Muleba		Ngara		District average*
		Freq	Prev (CI)	Freq	Prev (CI)	Freq	Prev (CI)	Freq	Prev (CI)	Freq	Prev (CI)	Freq	Prev (CI)	Freq	Prev (CI)	Prev
k13 P441L	2021	0/21	0.0 (0.0–16.1)	0/38	0.0 (0.0–9.3)	0/138	0.0 (0.0–2.6)	1/120	0.8 (0.0–4.6)	0/26	0.0 (0.0–13.2)	0/173	0.0 (0.0–2.1)	0/155	0.0 (0.0–2.4)	0.1
	2022	-	-	-	-	0/157	0.0 (0.0–2.3)	-	-	-	-	0/67	0.0 (0.0–5.4)	0/90	0.0 (0.0–4.0)	0
	2023	0/65	0.0 (0.0–5.5)	0/146	0.0 (0.0–2.5)	0/63	0.0 (0.0–5.7)	2/155	1.3 (0.2–4.6)	3/95	3.2 (0.7–9.0)	0/202	0.0 (0.0–1.8)	0/223	0.0 (0.0–1.6)	0.6
k13 F446I	2021	0/21	0.0 (0.0–16.1)	0/38	0.0 (0.0–9.3)	0/138	0.0 (0.0–2.6)	0/120	0.0 (0.0–3.0)	1/26	3.8 (0.1–19.6)	0/173	0.0 (0.0–2.1)	0/155	0.0 (0.0–2.4)	0.5
	2022	-	-	-	-	0/157	0.0 (0.0–2.3)	-	-	-	-	0/67	0.0 (0.0–5.4)	0/90	0.0 (0.0–4.0)	0
	2023	0/65	0.0 (0.0–5.5)	0/146	0.0 (0.0–2.5)	0/63	0.0 (0.0–5.7)	0/155	0.0 (0.0–2.4)	0/95	0.0 (0.0–3.8)	0/202	0.0 (0.0–1.8)	0/223	0.0 (0.0–1.6)	0
k13 G449A	2021	0/21	0.0 (0.0–16.1)	0/38	0.0 (0.0–9.3)	0/138	0.0 (0.0–2.6)	0/120	0.0 (0.0–3.0)	0/26	0.0 (0.0–13.2)	0/173	0.0 (0.0–2.1)	0/155	0.0 (0.0–2.4)	0
	2022	-	-	-	-	0/157	0.0 (0.0–2.3)	-	-	-	-	0/67	0.0 (0.0–5.4)	0/90	0.0 (0.0–4.0)	0
	2023	0/65	0.0 (0.0–5.5)	0/146	0.0 (0.0–2.5)	0/63	0.0 (0.0–5.7)	6/155	3.9 (1.4–8.2)	0/95	0.0 (0.0–3.8)	0/202	0.0 (0.0–1.8)	0/223	0.0 (0.0–1.6)	0.6
k13 C469Y	2021	0/21	0.0 (0.0–16.1)	0/38	0.0 (0.0–9.3)	0/138	0.0 (0.0–2.6)	0/120	0.0 (0.0–3.0)	0/26	0.0 (0.0–13.2)	0/173	0.0 (0.0–2.1)	0/155	0.0 (0.0–2.4)	0
	2022	-	-	-	-	0/157	0.0 (0.0–2.3)	-	-	-	-	0/67	0.0 (0.0–5.4)	0/90	0.0 (0.0–4.0)	0
	2023	0/65	0.0 (0.0–5.5)	0/146	0.0 (0.0–2.5)	1/63	1.6 (0.0–8.5)	0/155	0.0 (0.0–2.4)	0/95	0.0 (0.0–3.8)	0/202	0.0 (0.0–1.8)	0/223	0.0 (0.0–1.6)	0.2
k13 P527H	2021	0/21	0.0 (0.0–16.1)	1/39	2.6 (0.1–13.5)	0/146	0.0 (0.0–2.5)	0/132	0.0 (0.0–2.8)	0/24	0.0 (0.0–14.2)	0/184	0.0 (0.0–2.0)	0/174	0.0 (0.0–2.1)	0.4
	2022	-	-	-	-	0/170	0.0 (0.0–2.1)	-	-	-	-	0/82	0.0 (0.0–4.4)	1/96	1.0 (0.0–5.7)	0.3
	2023	0/74	0.0 (0.0–4.9)	0/155	0.0 (0.0–2.4)	0/66	0.0 (0.0–5.4)	0/170	0.0 (0.0–2.1)	0/104	0.0 (0.0–3.5)	0/229	0.0 (0.0–1.6)	0/251	0.0 (0.0–1.5)	0
k13 N537D	2021	0/21	0.0 (0.0–16.1)	0/39	0.0 (0.0–9.0)	0/146	0.0 (0.0–2.5)	0/132	0.0 (0.0–2.8)	0/24	0.0 (0.0–14.2)	0/184	0.0 (0.0–2.0)	0/174	0.0 (0.0–2.1)	0
	2022	-	-	-	-	0/170	0.0 (0.0–2.1)	-	-	-	-	0/82	0.0 (0.0–4.4)	0/96	0.0 (0.0–3.8)	0
	2023	0/74	0.0 (0.0–4.9)	0/155	0.0 (0.0–2.4)	0/66	0.0 (0.0–5.4)	1/170	0.6 (0.0–3.2)	0/104	0.0 (0.0–3.5)	0/229	0.0 (0.0–1.6)	0/251	0.0 (0.0–1.5)	0.1
k13 N537I	2021	0/21	0.0 (0.0–16.1)	0/39	0.0 (0.0–9.0)	0/146	0.0 (0.0–2.5)	0/132	0.0 (0.0–2.8)	0/24	0.0 (0.0–14.2)	0/184	0.0 (0.0–2.0)	0/174	0.0 (0.0–2.1)	0
	2022	-	-	-	-	0/170	0.0 (0.0–2.1)	-	-	-	-	0/82	0.0 (0.0–4.4)	0/96	0.0 (0.0–3.8)	0
	2023	0/74	0.0 (0.0–4.9)	0/155	0.0 (0.0–2.4)	0/66	0.0 (0.0–5.4)	0/170	0.0 (0.0–2.1)	0/104	0.0 (0.0–3.5)	1/229	0.4 (0.0–2.4)	0/251	0.0 (0.0–1.5)	0.1
k13 G538V	2021	0/21	0.0 (0.0–16.1)	0/39	0.0 (0.0–9.0)	0/146	0.0 (0.0–2.5)	0/132	0.0 (0.0–2.8)	0/24	0.0 (0.0–14.2)	0/184	0.0 (0.0–2.0)	0/174	0.0 (0.0–2.1)	0
	2022	-	-	-	-	0/170	0.0 (0.0–2.1)	-	-	-	-	0/82	0.0 (0.0–4.4)	0/96	0.0 (0.0–3.8)	0
	2023	0/74	0.0 (0.0–4.9)	0/155	0.0 (0.0–2.4)	0/66	0.0 (0.0–5.4)	0/170	0.0 (0.0–2.1)	0/104	0.0 (0.0–3.5)	1/229	0.4 (0.0–2.4)	0/251	0.0 (0.0–1.5)	0.1
k13 I543T	2021	0/21	0.0 (0.0–16.1)	0/39	0.0 (0.0–9.0)	0/146	0.0 (0.0–2.5)	0/132	0.0 (0.0–2.8)	0/24	0.0 (0.0–14.2)	0/184	0.0 (0.0–2.0)	0/174	0.0 (0.0–2.1)	0
	2022	-	-	-	-	0/170	0.0 (0.0–2.1)	-	-	-	-	0/82	0.0 (0.0–4.4)	0/96	0.0 (0.0–3.8)	0
	2023	0/74	0.0 (0.0–4.9)	1/155	0.6 (0.0–3.5)	0/66	0.0 (0.0–5.4)	1/170	0.6 (0.0–3.2)	0/104	0.0 (0.0–3.5)	0/229	0.0 (0.0–1.6)	0/251	0.0 (0.0–1.5)	0.2
k13 R561H	2021	0/21	0.0 (0.0–16.1)	0/35	0.0 (0.0–10.0)	31/136	22.8 (16.0–30.8)	17/118	14.4 (8.6–22.1)	0/24	0.0 (0.0–14.2)	0/171	0.0 (0.0–2.1)	2/144	1.4 (0.2–4.9)	5.5
	2022	-	-	-	-	37/162	22.8 (16.6–30.1)	-	-	-	-	7/70	10.0 (4.1–19.5)	1/83	1.2 (0.0–6.5)	11.3
	2023	0/70	0.0 (0.0–5.1)	1/147	0.7 (0.0–3.7)	16/62	25.8 (15.5–38.5)	22/158	13.9 (8.9–20.3)	0/98	0.0 (0.0–3.7)	11/220	5.0 (2.5–8.8)	7/236	3.0 (1.2–6.0)	6.9
k13 V568G	2021	0/21	0.0 (0.0–16.1)	2/35	5.7 (0.7–19.2)	0/136	0.0 (0.0–2.7)	1/118	0.8 (0.0–4.6)	0/24	0.0 (0.0–14.2)	0/171	0.0 (0.0–2.1)	1/144	0.7 (0.0–3.8)	1
	2022	-	-	-	-	0/162	0.0 (0.0–2.3)	-	-	-	-	0/70	0.0 (0.0–5.1)	1/83	1.2 (0.0–6.5)	0.4
	2023	0/70	0.0 (0.0–5.1)	1/147	0.7 (0.0–3.7)	0/62	0.0 (0.0–5.8)	0/158	0.0 (0.0–2.3)	0/98	0.0 (0.0–3.7)	0/220	0.0 (0.0–1.7)	0/236	0.0 (0.0–1.6)	0.1
k13 P574L	2021	0/21	0.0 (0.0–16.1)	0/35	0.0 (0.0–10.0)	0/136	0.0 (0.0–2.7)	0/118	0.0 (0.0–3.1)	0/24	0.0 (0.0–14.2)	0/171	0.0 (0.0–2.1)	0/144	0.0 (0.0–2.5)	0
	2022	-	-	-	-	0/162	0.0 (0.0–2.3)	-	-	-	-	0/70	0.0 (0.0–5.1)	1/83	1.2 (0.0–6.5)	0.4
	2023	0/70	0.0 (0.0–5.1)	0/147	0.0 (0.0–2.5)	0/62	0.0 (0.0–5.8)	0/158	0.0 (0.0–2.3)	0/98	0.0 (0.0–3.7)	1/220	0.5 (0.0–2.5)	1/236	0.4 (0.0–2.3)	0.1
k13 R622I	2021	0/21	0.0 (0.0–16.1)	0/39	0.0 (0.0–9.0)	0/148	0.0 (0.0–2.5)	0/127	0.0 (0.0–2.9)	0/27	0.0 (0.0–12.8)	0/183	0.0 (0.0–2.0)	0/168	0.0 (0.0–2.2)	0
	2022	-	-	-	-	0/166	0.0 (0.0–2.2)	-	-	-	-	0/84	0.0 (0.0–4.3)	0/99	0.0 (0.0–3.7)	0
	2023	0/70	0.0 (0.0–5.1)	0/153	0.0 (0.0–2.4)	0/65	0.0 (0.0–5.5)	0/162	0.0 (0.0–2.3)	1/101	1.0 (0.0–5.4)	0/216	0.0 (0.0–1.7)	0/242	0.0 (0.0–1.5)	0.1
k13 A675V	2021	0/21	0.0 (0.0–16.1)	0/41	0.0 (0.0–8.6)	1/138	0.7 (0.0–4.0)	0/122	0.0 (0.0–3.0)	0/26	0.0 (0.0–13.2)	0/173	0.0 (0.0–2.1)	0/162	0.0 (0.0–2.3)	0.1
	2022	-	-	-	-	1/170	0.6 (0.0–3.2)	-	-	-	-	0/86	0.0 (0.0–4.2)	0/94	0.0 (0.0–3.8)	0.2
	2023	0/66	0.0 (0.0–5.4)	0/143	0.0 (0.0–2.5)	3/62	4.8 (1.0–13.5)	1/153	0.7 (0.0–3.6)	0/97	0.0 (0.0–3.7)	1/199	0.5 (0.0–2.8)	0/224	0.0 (0.0–1.6)	0.9

The data presented is the number of samples containing mutant alleles over the total number successfully genotyped (obs/pop), the calculated prevalence, expressed as a percentage (prev), and the 95% confidence interval for the prevalence as determined by the exact (also known as beta) approximation interval method, as implemented by the Python statsmodels module (v.0.14.4), also expressed as a percentage (CI).

* District Average is the equally weighted average percentage of the districts for the region.

### Changes in mutations associated with tolerance to partner drugs and anti-folates

**FIGURE 2:**
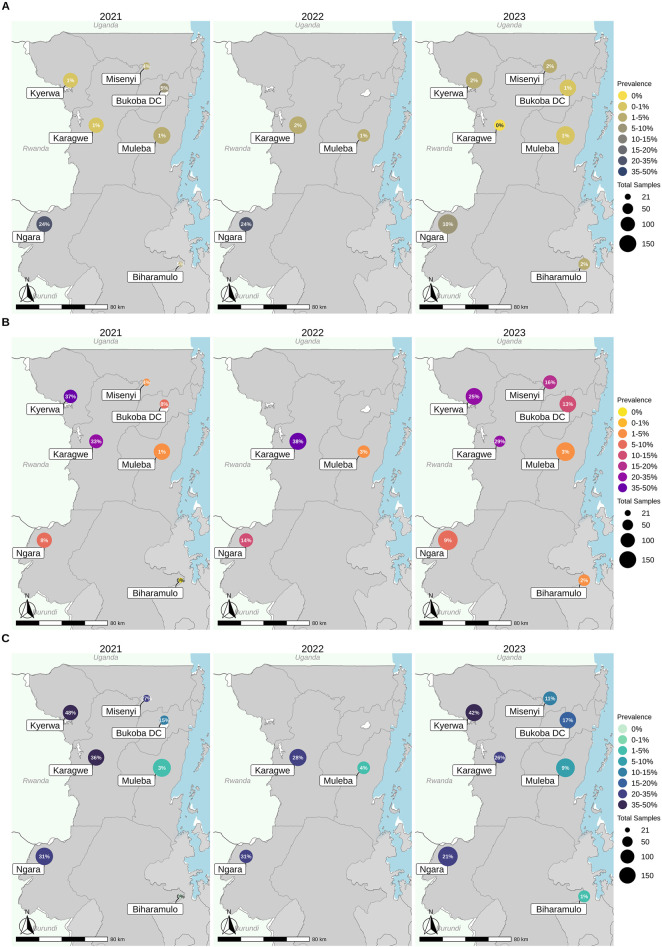
Key Mutations for chloroquine and sulfadoxine-pyremethamine resistance and their spatio-temporal correlation. Drug resistance mutations are shown across years and sites. The chloroquine resistance mutation CRT 76T is shown in **(A)**. The pyremethamine resistance mutation DHFR 164L **(B)**, and sulfadoxine resistance mutation DHPS 581G **(C)**, are also shown. Prevalences are shown as percentages along with the underlying sample counts.

Overall, the changes in partner drug associated mutations were minimal. The MDR1 N86Y mutation is associated with increased susceptibility to lumefantrine and decreased susceptibility to amodiaquine (Malmberg et al., 2013). The MDR1 86Y mutation has very low prevalence in all districts across the years (Table 2) consistent with the predominance of AL therapy selecting for the wild type allele in Tanzania. We also analyzed the NFD haplotype (MDR1 N86, 184F, and D1246) and found high prevalences of this haplotype in several districts (Supplementary Table S4). Because the NFD haplotype consists of wildtype N86 and wildtype D1246, the NFD haplotype can be alternatively thought of as a single MDR1 Y184F mutation. This mutation increased in prevalence from 40.8% to 47.2% from 2021 to 2023 (year-over-year OR: 1.10, CI [0.99, 1.21] (Supplementary Table S5). Overall, the K76T mutant allele of CRT, associated with reduced amodiaquine susceptibility and increased lumefantrine susceptibility (Olliaro et al., 1996), occurred at a low prevalence, with a decrease from 5.9% in 2021 to 2.4% in 2023 (annual odds ratio 0.63; 95% CI [0.49, 0.80]) (Table 2). This was primarily driven by a decrease in Ngara, which decreased from 24.2% (CI: 17.1%–32.6%) to 9.7% (CI: 6.1%–14.5%) over this period (Fisher’s exact test, *p = 0.00051;* corrected *p = 0.0036)* (Figure 2A).

**TABLE 2:** District level prevalence of partner drug (MDR1 and CRT), DHFR and DHPS mutations of interest.

Mutation	Year	Biharamulo		Bukoba DC		Karagwe		Kyerwa		Misenyi		Muleba		Ngara		District average*
		Freq	Prev (CI)	Freq	Prev (CI)	Freq	Prev (CI)	Freq	Prev (CI)	Freq	Prev (CI)	Freq	Prev (CI)	Freq	Prev (CI)	Prev
crt K76T	2021	1/20	5.0 (0.1–24.9)	2/38	5.3 (0.6–17.7)	1/116	0.9 (0.0–4.7)	1/110	0.9 (0.0–5.0)	1/25	4.0 (0.1–20.4)	2/152	1.3 (0.2–4.7)	31/128	24.2 (17.1–32.6)	5.9
	2022	-	-	-	-	3/164	1.8 (0.4–5.3)	-	-	-	-	1/76	1.3 (0.0–7.1)	23/94	24.5 (16.2–34.4)	9.2
	2023	1/61	1.6 (0.0–8.8)	1/144	0.7 (0.0–3.8)	0/56	0.0 (0.0–6.4)	3/144	2.1 (0.4–6.0)	2/94	2.1 (0.3–7.5)	1/190	0.5 (0.0–2.9)	21/216	9.7 (6.1–14.5)	2.4
dhfr I164L	2021	0/20	0.0 (0.0–16.8)	3/37	8.1 (1.7–21.9)	31/95	32.6 (23.4–43.0)	33/89	37.1 (27.1–48.0)	1/25	4.0 (0.1–20.4)	2/142	1.4 (0.2–5.0)	10/118	8.5 (4.1–15.0)	13.1
	2022	-	-	-	-	59/155	38.1 (30.4–46.2)	-	-	-	-	2/73	2.7 (0.3–9.5)	13/94	13.8 (7.6–22.5)	18.2
	2023	1/62	1.6 (0.0–8.7)	19/147	12.9 (8.0–19.4)	17/58	29.3 (18.1–42.7)	37/150	24.7 (18.0–32.4)	15/95	15.8 (9.1–24.7)	5/194	2.6 (0.8–5.9)	20/219	9.1 (5.7–13.8)	13.7
dhps A581G	2021	0/21	0.0 (0.0–16.1)	6/40	15.0 (5.7–29.8)	54/150	36.0 (28.3–44.2)	62/129	48.1 (39.2–57.0)	7/26	26.9 (11.6–47.8)	6/190	3.2 (1.2–6.7)	55/177	31.1 (24.3–38.5)	22.9
	2022	-	-	-	-	47/168	28.0 (21.3–35.4)	-	-	-	-	3/81	3.7 (0.8–10.4)	28/91	30.8 (21.5–41.3)	20.8
	2023	1/70	1.4 (0.0–7.7)	26/153	17.0 (11.4–23.9)	17/65	26.2 (16.0–38.5)	69/166	41.6 (34.0–49.5)	11/100	11.0 (5.6–18.8)	18/209	8.6 (5.2–13.3)	49/235	20.9 (15.8–26.6)	18.1
mdr1 N86Y	2021	0/20	0.0 (0.0–16.8)	0/38	0.0 (0.0–9.3)	0/148	0.0 (0.0–2.5)	1/121	0.8 (0.0–4.5)	0/26	0.0 (0.0–13.2)	0/180	0.0 (0.0–2.0)	2/179	1.1 (0.1–4.0)	0.3
	2022	-	-	-	-	0/155	0.0 (0.0–2.4)	-	-	-	-	0/64	0.0 (0.0–5.6)	0/79	0.0 (0.0–4.6)	0
	2023	0/65	0.0 (0.0–5.5)	1/141	0.7 (0.0–3.9)	0/60	0.0 (0.0–6.0)	0/150	0.0 (0.0–2.4)	0/96	0.0 (0.0–3.8)	0/191	0.0 (0.0–1.9)	1/215	0.5 (0.0–2.6)	0.2

# The data presented is the number of samples containing mutant alleles over the total number successfully genotyped (obs/pop), the calculated prevalence, expressed as a percentage (prev), and the 95% confidence interval for the prevalence as determined by the exact (also known as beta) approximation interval method, as implemented by the Python statsmodels module (v.0.14.4), also expressed as a percentage (CI).

* District Average is the equally weighted average of the districts for the region.

Antifolate resistance mutations also saw small spatial and temporal shifts between 2021 and 2023. Mutations in DHFR are associated with resistance to pyrimethamine and are acquired sequentially. The older DHFR mutations 51I, 59R, and 108N were all near fixation in all districts and all years surveyed, while the recent 164L mutation, which confers high-level pyrimethamine resistance as well as resistance to dapsone, showed more variable patterns of prevalence across districts and years, ranging from completely absent in Biharamulo in 2021 to 38.1% in Karagwe in 2022. The overall pattern was an East to West gradient, with the mutation at higher prevalence in four of the Western sites near Rwanda (Figure 2B), but low in three Eastern sites near Lake Victoria (temporal trends not significant). Linear modeling revealed no overall increase in the 164L mutation across Kagera (year-over-year OR 0.96, CI [0.81, 1.13]). Sequential mutations in DHPS are associated with resistance to sulfadoxine, with high-level resistance conferred by three mutations (437G, 540E, and 581G). The older DHPS 437G and 540E were both present near fixation across all districts and years surveyed, while the latest mutation, DHPS 581G, showed an East to West gradient similar to DHFR-164L (Figure 2C), with district-level prevalences ranging from completely absent in Biharamulo in 2021, to 48.1% prevalence in Kyerwa in 2021 (regional linear model: year-over-year OR 0.86, CI [0.76, 0.98]). District-level frequencies of DHPS 581G and DHFR 164L mutations were significantly correlated with each other (weighted Spearman *R* ^2^ = 0.58, P = 0.045, Supplementary Figure S3). We observed a year-over-year unweighted average of 5.5% of samples carrying both DHPS 581G and DHFR 164L; when filtering to samples with homozygous calls, 3.5% of parasite genomes contained both drug-resistance alleles. (Supplementary Table S4). A full list of prevalences, coverages, and confidence intervals for known key drug resistance point mutations can be found in Supplementary Table S5.

MIP probe UMI counts were additionally analyzed to assess prevalence of copy number variation (CNV). We normalized MIP UMI counts, generating a transformed gene copy count distribution centered at 1 for the haploid malaria genome. With this normalization, we expect to detect potential CNV as a multi-modal distributions with peaks centered at higher integer gene counts. We observed generally tight, unimodel distributions centered around this modal copy number, with the exception of *plasmepsin3 and crt* in 2023 samples, which normalized poorly due to variable probe performance. Setting a conservative threshold of a normalized gene copy count of 1.75, we identify no definitive evidence of CNV in major drug resistance genes over the study period (Supplementary Figure S4).

## Discussion

Between 2021 and 2023, malaria molecular surveillance (MMS) in the Kagera region revealed a concerning but nuanced picture of emerging ART-R. The WHO-validated K13 mutation R561H remains the predominant marker of ART-R, with evidence of both persistence in the original hotspot along the Rwanda border and gradual expansion eastward. Across 2,866 successfully genotyped isolates from seven districts the average R561H prevalence rose from 5.5% in 2021 to 6.9% in 2023. The highest and most consistent prevalence occurred in Karagwe (22.8%–25.8%) and Kyerwa (14.4%–13.9%) (trends not significant), confirming sustained transmission in districts bordering Rwanda. Importantly, R561H was detected for the first time in Muleba (10.0%) and Bukoba (0.7%) in 2022 and 2023, respectively, with Ngara showing a modest statistically insignificant rise (1.4% → 3.0%), indicating eastward spread into new districts. While the overall prevalence remains relatively low, any geographic expansion of K13 mutations warrants close attention, and it is somewhat reassuring that prevalence did not escalate further and did not involve additional districts over the two-year period.

Other validated K13 mutations also demonstrated early signals of spatial diversification. A675V, initially restricted to Karagwe, emerged in Muleba and Kyerwa by 2023. V568G appeared sporadically in Bukoba and Kyerwa, P441L emerged in Kyerwa and Misenyi, and the Horn-of-Africa–associated R622I mutation was detected once in Misenyi. Candidate and non-validated polymorphisms remained rare but increased slightly in frequency and numbers in later years, especially in Karagwe and Ngara. Collectively, these patterns indicate a parasite population undergoing continued diversification under local selection pressures.

Markers associated with ACT partner-drugs displayed more modest temporal shifts consistent with ACT usage. AL has been the first-line treatment for uncomplicated *Plasmodium falciparum* malaria since 2006, resulting in sustained lumefantrine drug pressure across the country. The pfmdr1 N86Y mutation, which has been associated with decreased susceptibility to amodiaquine and increased susceptibility to lumefantrine, was uniformly low, or nearly fixed as the wildtype allele across all districts in this study. The *pfmdr1* **NFD** haplotype (N86, 184F, D1246) is frequently reported in parasite populations in regions of Africa with extensive use of AL. *In vitro* functional studies suggest that *pfmdr1* variants, particularly at codon 86, can modulate lumefantrine susceptibility through altered transporter activity. The independent contributions of 184F and D1246 appear to be limited, consistent with our observed near fixation of N86 but not 184F (Veiga et al., 2016; Shafik et al., 2022). *In vitro* editing of multicopy Dd2 found that N86-184F appears to be more tolerant than N86-Y184 (Calçada et al., 2020). If N86-184F is slightly more tolerant, with an effect more appreciable in multicopy strains, that could account for its rise from 40.8% to 47.2% (Supplementary Table S5) during the study period, but further study is needed, particularly given that duplications of mdr1 have not been observed at any appreciable level in Africa.

Similarly, the pfcrt K76T mutation, historically selected under chloroquine pressure and associated with amodiaquine resistance, declined from 5.9% (2021) to 2.4% (2023), largely driven by decreasing prevalence in Ngara (24.2%–9.7%). The functional role of pfcrt K76T in modulating chloroquine and related drug responses has been well established experimentally (Venkatesan et al., 2014; Mwai et al., 2009). In addition, emerging evidence indicates that pfcrt variation can influence lumefantrine susceptibility, with the wildtype K76 allele being selected in AL-treated populations, further supporting the role of lumefantrine-driven selection in shaping parasite population structure (Sisowath et al., 2005).

Antifolate resistance remains entrenched: early DHFR mutations (N51I, C59R, S108N) approached fixation, while high-level resistance markers associated with sulfadoxine-pyrimethamine resistance DHPS A581G and DHFR I164L, respectively exhibited substantial spatial and temporal heterogeneity. DHFR I164L ranged from 0% in Biharamulo in 2021 to 38.1% in Karagwe in 2022, while DHPS A581G peaked at 48.1% in Kyerwa. Their correlation (Supplementary Figure S3 weighted Spearman’s *r* ^2^ = 0.58, P = 0.045) suggests co-selection of high-level SP resistance haplotypes, a critical concern in regions implementing intermittent preventive treatment in pregnancy (IPTp). Tanzania routinely implements intermittent preventive treatment in pregnancy (IPTp) in regions of high and moderate transmission, including Kagera. The anti-folate sulfadoxine-pyrimethamine (SP) remains the key drug for IPTp. A recent meta-analysis indicated that IPTp with SP is associated with improved birth outcomes even when DHPS K540E is >90% but not when DHPS A581G is >10% (van Eijk et al., 2019).

The spatial distribution of these mutations follows a clear west-to-east gradient, with both DHFR I164L and DHPS A581G more prevalent in western sites near Rwanda and Uganda, and declining toward eastern sites (Figure 2). This pattern aligns with recent findings from genomic surveillance studies in Rwanda (Wernsman Young et al., 2025) and Uganda (Katairo et al., 2026), where high frequencies of DHPS A581G and emerging DHFR I164L have been reported, suggesting that this region may serve as a reservoir or source of highly resistant parasite lineages. These cross-border dynamics likely reflect a combination of human mobility, shared transmission corridors around Lake Victoria, and similar drug selection pressures.

The West-East gradient observed here is also quite pronounced; DHFR I164L and DHPS A581G ranged from near zero in Eastern districts to over 40% in Western districts near Rwanda and Uganda. These stark gradients underscore that resistance is shaped by highly localized factors rather than uniform regional pressures. Test positivity rate can be used as a proxy for transmission intensity, noting that this correlation is limited in the districts with the highest and lowest transmission intensity (Okiring et al., 2021). Transmission intensity is one likely driver: although six of seven clinics were situated in moderate-to-high transmission districts (Petro et al., 2024, average RDT test positivity ≥20%), high transmission did not appear to suppress increases in ART-R mutations, contrary to some theoretical expectations regarding within-host competition. Local drug use patterns, stock variability, IPTp coverage, and human mobility patterns likely contribute to the localized emergence and persistence of resistant haplotypes.

Together, these findings across drug resistance markers reinforce the need for continued molecular surveillance across border regions. Spreading ART-R may act as a gateway to the emergence of ACT partner-drug resistance (Niare et al., 2025). As seen in Southeast Asia, delayed parasite clearance increases the probability that parasites are exposed to subtherapeutic concentrations of the partner drug, thereby widening the selection window for resistant variants. In the African context, where AL remains the first-line treatment in most endemic countries, this creates ideal conditions for the stepwise selection of lumefantrine-tolerant parasites. In Uganda, *ex vivo* susceptibility to lumefantrine, Africa’s dominant partner drug, declined between 2019 and 2024 (Okitwi et al., 2025), and returning travelers have shown treatment failures accompanied by decreased *in vitro* lumefantrine susceptibility (van Schalkwyk et al., 2024; Oliveira et al., 2025). Molecular data increasingly support this trajectory, with shifts in allele frequencies of key loci such as *pfmdr1* and *pfcrt* variants associated with reduced lumefantrine susceptibility being reported across East Africa, including in Rwanda and Uganda (Tumwebaze et al., 2022). These findings highlight the critical need to expand surveillance beyond artemisinin resistance markers alone and systematically monitor partner-drug susceptibility using integrated genomic, clinical, and pharmacological approaches. Early detection of shifts in reduced lumefantrine sensitivity, before widespread clinical failure occurs, will be essential to guide timely treatment policy adjustments and to prevent the establishment of fully resistant parasite populations in Africa.

This study also has important limitations. Sampling was uneven across years, with four districts not surveyed in 2022, limiting the resolution of temporal trends. To visualize the unevenness in the number of samples that had enough coverage to make inferences for each mutation across time, we display the denominators from k13-R561H, crt-76T, dhfr-164L, and dhps-581G as a line graph. (Supplementary Figure S1). Genotyping success varied by locus and year, potentially biasing prevalence estimates. The absence of whole-genome sequencing data prevents distinguishing whether R561H observations represent clonal expansion or multiple introductions. Sampling was limited to symptomatic clinic attendees, which may not fully represent community-level parasite diversity. The choice of thresholds (three UMIs of coverage and one UMI supporting an alternate allele for known mutations) can have a meaningful impact on inferred mutation prevalences, and while it is most appropriate to maximize sensitivity for known mutations while setting more conservative filters for novel mutations, when a conservative coverage threshold of ten and alternate threshold of three is used instead, some inferred key drug resistance mutation prevalences shift slightly (Supplementary Table S6).

The data reinforce that ART-R remains an emerging threat that must be monitored through continued surveillance. The TZ2 haplotype of R561H, first identified in Kagera in 2021, has since been detected in other regions of Tanzania, highlighting the potential for wider dissemination. Ongoing sequencing from all regions of Mainland Tanzania within the MSMT program will help clarify whether Kagera continues to seed R561H elsewhere. National malaria control programs in affected areas must therefore strengthen surveillance systems to ensure rapid detection and mitigation. Molecular markers of resistance, particularly K13 polymorphisms, are already being incorporated into modeling studies exploring the impact of alternative treatment strategies. Continued, granular MMS will be indispensable for informing these policy decisions and preserving ACT effectiveness across the region.

## Data Availability

The datasets presented in this study can be found in online repositories. The names of the repository/repositories and accession number(s) can be found below: https://www.ncbi.nlm.nih.gov/, PRJNA1090883.
