# Supplementary figures and images for: Temporal trends in artemisinin partial resistance and other antimalarial drug mutations in Plasmodium falciparum from Kagera region, Northwestern Tanzania, 2021–2023
Source: Front Genet. 2026 Jun 3;17:1776108. doi: 10.3389/fgene.2026.1776108 (PMC13271678; doi:10.3389/fgene.2026.1776108)

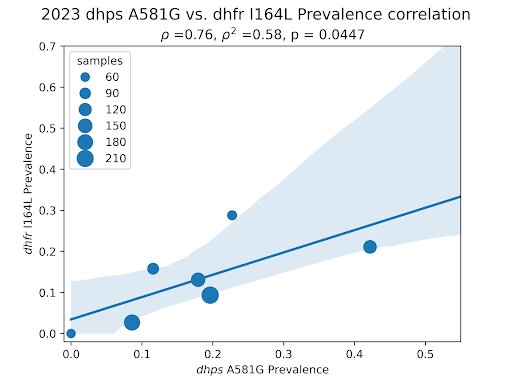

Supplement: Supplementary file 1 [file Image3.jpeg]

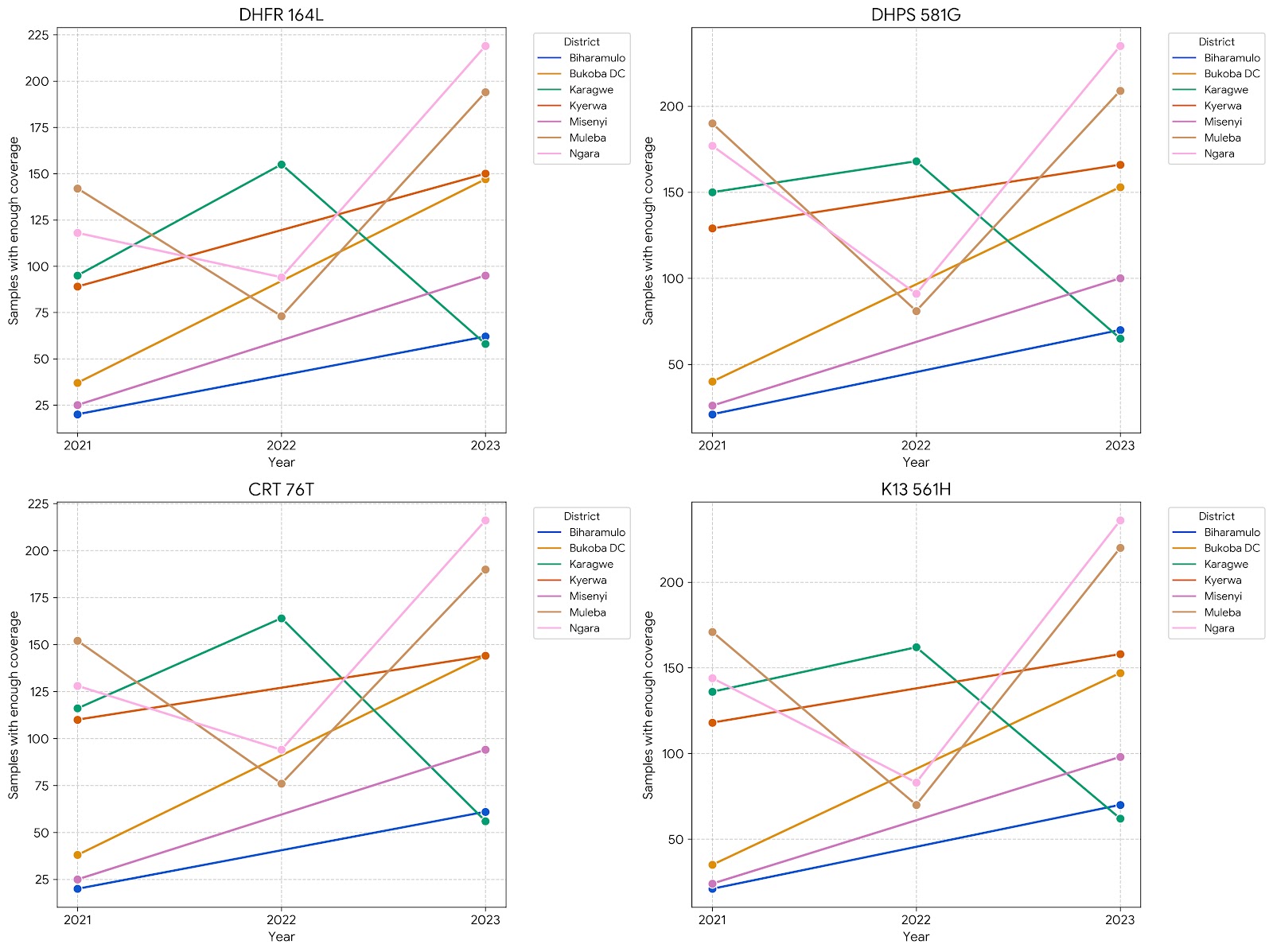

Supplement: Supplementary file 2 [file Image1.jpeg]

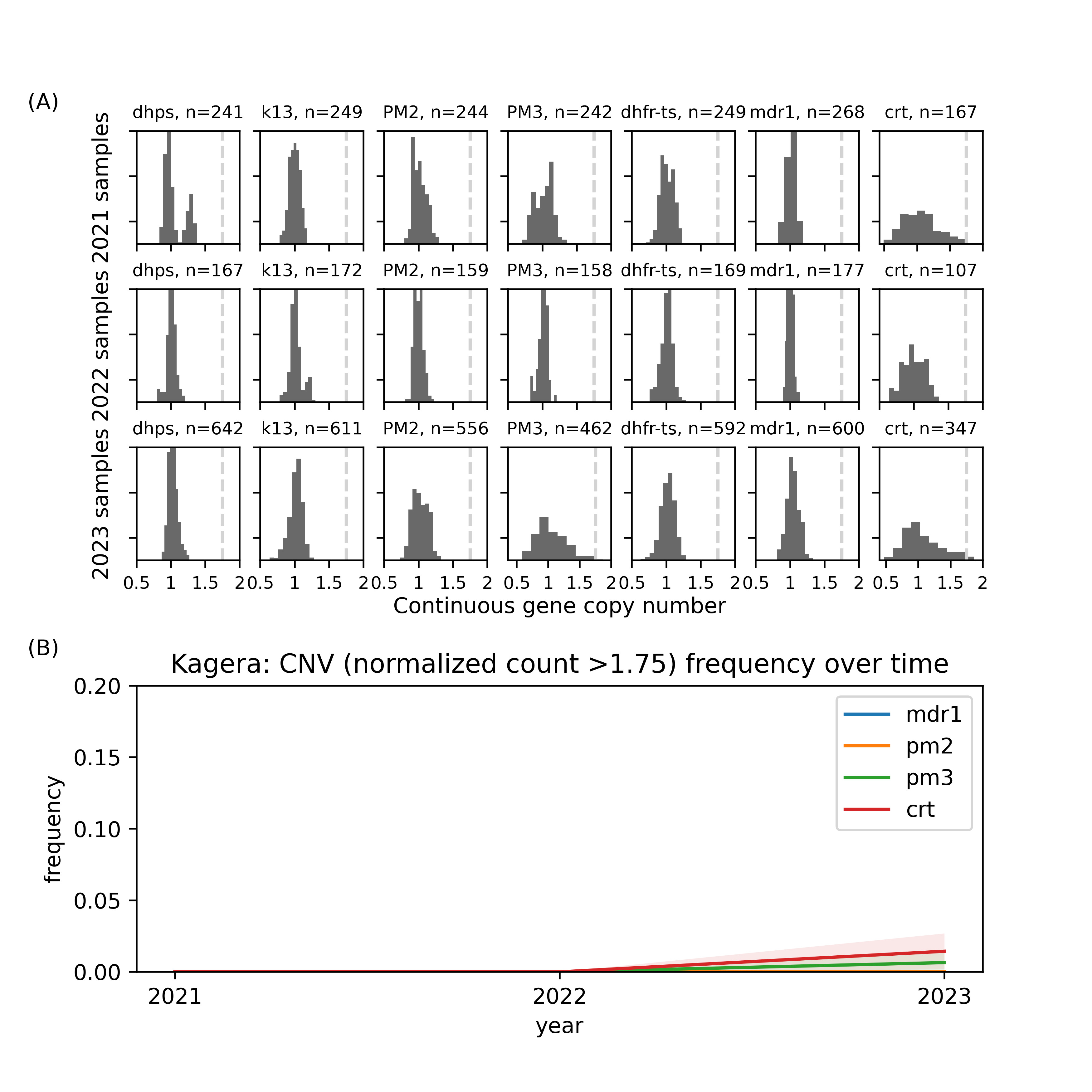

Supplement: Supplementary file 3 [file Image4.jpeg]

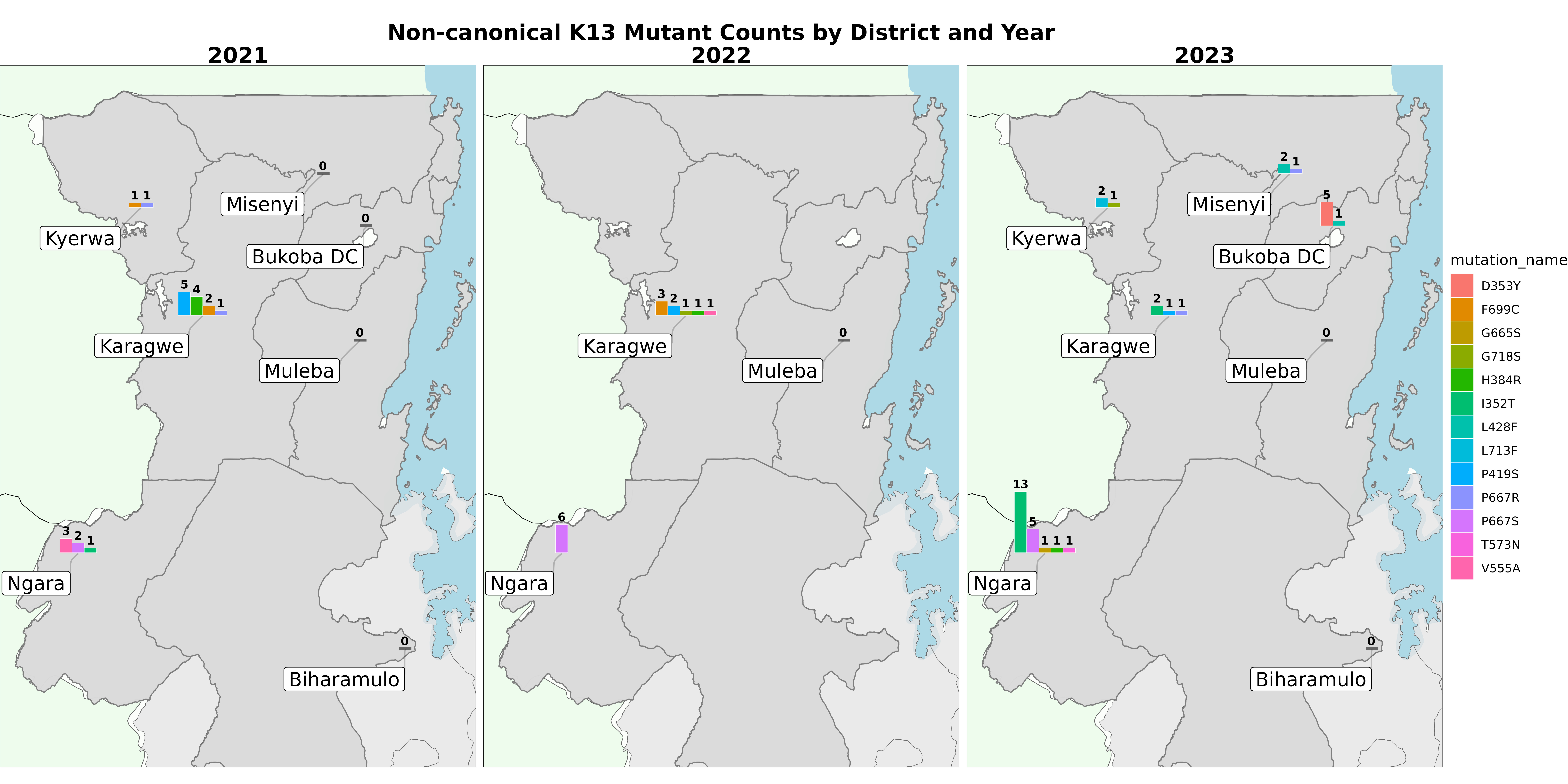

Supplement: Supplementary file 4 [file Image2.jpeg]
